# Povidone-iodine nasal spray (Nasodine^®^) for the common cold: a randomized, controlled, double-blind, Phase III clinical trial

**DOI:** 10.3389/fmed.2025.1565069

**Published:** 2025-06-05

**Authors:** Thomas M. Polasek, Peter L. Friedland

**Affiliations:** ^1^Centre for Medicine Use and Safety, Monash University, Melbourne, VIC, Australia; ^2^Medical School, University of Western Australia, Perth, WA, Australia

**Keywords:** povidone-iodine, upper respiratory tract infections (URTIs), Nasodine, nasal spray, common cold, clinical trial

## Abstract

**Aim:**

To determine the safety and efficacy of a 0.5% povidone-iodine nasal spray (Nasodine) as a treatment for the common cold (ACTRN12619000764134).

**Methods:**

A multi-center, randomized, controlled, double-blind Phase III study was conducted to assess the impact of Nasodine on the common cold. Two hundred and sixty (260) euthyroid adults with qualifying cold symptoms and meeting inclusion/exclusion criteria were randomized 2:1 to Nasodine or matching saline nasal spray (SNS), each applied 4 times daily for 5 days. Cold severity was reported using the WURSS-21 survey. The primary endpoint was impact on nasal symptoms (4-item scale), with the validated 19-item Global Severity Score (GSS) as the key secondary endpoint.

**Results:**

All cold severity outcomes pointed in favor of Nasodine over SNS. In the ITT (*n* = 260), the Nasodine benefit over SNS on nasal symptoms was 8.4% (*p* = 0.217). For GSS, the benefit was 12.6% (*p* = 0.054) in the ITT population. *Post hoc* subset analyses showed markedly improved benefits of Nasodine: In subjects with stronger symptoms at enrollment (ES), the GSS benefit was 17.1% (*p* = 0.023); for those with confirmed viral infection (VES), GSS benefit was 23.0% (*p* = 0.048); and for those enrolled within 24 h of symptom onset (24S), GSS benefit was 39.7% (*p* = 0.024). In terms of functional impairment, the Nasodine benefit was greater in all subsets, with 16.1% (*p* = 0.041) benefit in ITT, 22.2% in ES (*p* = 0.012), 32.1% in VES (*p* = 0.023) and 37.1% in 24S (*p* = 0.093). Nasodine was well tolerated, with mild transient nasopharyngeal discomfort being a common adverse effect.

**Conclusion:**

Nasodine treatment had a consistently positive and clinically meaningful benefit on overall cold severity when compared with saline nasal spray. Early treatment after symptom onset is an important efficacy factor.

**Clinical trial registration:**

https://www.anzctr.org.au/Trial/Registration/TrialReview.aspx?id=377353&isReview=true, identifier ACTRN12619000764134.

## Introduction

Povidone-iodine (PVP-I) is a broad-spectrum topical microbicide that rapidly inactivates viruses and bacteria at low concentrations and has no history of microbial resistance (1–3). PVP-I has been used as a skin antiseptic since 1955 and has been on the World Health Organization (WHO) *List of Essential Medicines* since 1993 (4).

PVP-I is a complex of povidone (PVP), a polymer, and molecular iodine. The polymer solubilizes molecular iodine by holding the available iodine within the hydrophobic core of the highly coiled PVP molecule, and only a small fraction of the available iodine in the aqueous phase as “free” iodine (I_2_), which is the microbially active chemical moiety (5, 6). Formulations for skin and wound antisepsis typically employ PVP-I concentrations of 5–10% w/v. The antimicrobial potency of PVP-I solutions increases as the PVP-I concentration decreases, with maximum microbicidal potency occurring in the range 0.1–1.0% w/v. This is due to the release of higher concentrations of free iodine as the PVP-I complex is diluted. As a result, 0.5–1.0% PVP-I solutions have higher immediate antimicrobial potency than more concentrated PVP-I solutions, although they have reduced antimicrobial “capacity” due to the diminished iodine reserve retained by the polymer (5, 7). Low concentration PVP-I solutions (<1.0%) also have reduced potential for adverse effects, which are primarily related to the PVP-I concentration (6), and have reduced potential for systemic iodine absorption because of the lower total iodine present. The combination of high potency and low adverse effects makes low concentration PVP-I solutions well-suited for use on human mucous membranes as topical microbicides.

Formulations of PVP-I for oral use as a gargle or mouthwash have been available for about forty years and are widely used worldwide. These typically employ concentrations in the range 0.5–1.0% w/v. Using PVP-I in the nose is a more recent innovation and one subject to greater safety scrutiny given the highly absorptive nature of the nasal passages and the potential for ciliotoxicity. One study (8) reported that 0.08% PVP-I nasal rinses resulted in significant reductions in signs of infection and notable symptom benefits in subjects with recalcitrant chronic rhinosinusitis, with no impact on thyroid function, mucociliary clearance or olfaction. During the COVID-19 pandemic, there was experimental use of PVP-I nasal irrigation and nasal sprays for reduction of viral shedding and treatment of the illness. Trials commonly employed concentrations of 0.4–0.6% w/v PVP-I (8–11). Studies were of varying evidentiary quality, although several reported viral shedding benefits and none reported significant adverse effects. One recent randomized controlled Phase II study in COVID-19 patients (12) assessed the impact on viral shedding using a 0.5% w/v PVP-I nasal spray (Nasodine^®^) that was applied eight times daily for two and a half days. The treatment produced a statistically significant reduction versus placebo in viral shedding from the nasal passages, with 100% clearance of viable virus from nasal swabs by the fourth day. Despite the reported activity of PVP-I in COVID-19 and its known virucidal activity against other respiratory viruses, the potential for intranasal PVP-I in the management of upper respiratory infections (URTIs), such as the common cold, has not been investigated.

The common cold is a symptom complex initiated by an infection of the epithelial cells inside the nasal cavity and is mostly caused by viruses, with up to 200 antigenically distinct viruses from eight different genera known to cause colds. The most common cause of colds are the rhinoviruses, which are responsible for around 50% of adult colds, with the second most common cause being seasonal coronaviruses; colds can also be caused by influenza viruses, RSV, adenoviruses, metapneumovirus and parainfluenza viruses (13). In approximately 30% of people with cold symptoms in the natural setting, the causative organism cannot be identified, although a viral cause is likely (13). Despite the range of infective causes, the symptoms of the clinical condition that result from the infection are similar. Symptoms can include sneezing, rhinorrhea, nasal congestion, sore throat, coughing, headache, malaise, myalgia and chills/fever. It is the constellation of these symptoms (the symptom complex) that defines the condition known as the common cold, rather than the infecting organism.

While URTIs are generally mild and self-resolving illnesses that cause little mortality, they have high incidence and infectivity and contribute to significant morbidity and high economic losses due to lost productivity (14, 15). Such costs include symptomatic medications, doctor visits and unnecessary antibiotic prescriptions. In vulnerable populations, such as asthmatics, the immune-compromised and those with chronic lung diseases, a URTI can trigger serious disease exacerbations or lead to more serious lower respiratory infections with attendant risks of hospitalization and death (16). As the reporting definition of URTI excludes otitis media and all lower respiratory infections (14), the term “URTI” can be considered virtually coincident with the “common cold” and the two terms are often used interchangeably in the literature.

In 2019, it was estimated that there were 17.2 billion URTI worldwide, making URTI by far the most common illness of humanity, with its incidence almost 3 times greater than the next most common disease group (diarrheal diseases) and 35 times greater than all lower respiratory tract infections combined (14). In that study, the disease burden measured as disability adjusted life years (DALYs) associated with URTI in North America, was 379,300 DALY in 2019. In another study, the value of one DALY averted in very high HDI (human development index) countries like USA and Canada was estimated at US$69,499 (17), suggesting a total annual cost of around $26 billion in North America alone. This is despite the availability of all existing medications for treating colds. Given the scale, disease burden and cost of URTIs, any new approach to treatment that can deliver even a modest impact on symptoms and functional impairment could produce a large overall benefit.

The potential clinical use of intranasal PVP-I at low concentrations for the treatment of URTI was first hypothesized in 2015 (18). The authors proposed that, although PVP-I acts topically and is not absorbed into nasal cells where viral replication may be occurring, frequent use of a PVP-I nasal spray could sufficiently suppress the extracellular viral load to interrupt the infection cycle and thereby reduce URTI severity and duration. In addition to the direct virucidal mechanism, PVP-I could affect the binding of the virus to cellular receptor proteins and inactivate immune signaling proteins that propagate symptomatology, both being mechanisms previously reported in the literature for PVP-I (19, 20).

Nasodine^®^ Nasal Spray (“Nasodine”) is a commercially available 0.5% w/v PVP-I nasal spray that has undergone significant development, including pharmaceutical packaging development, GMP manufacturing, preclinical testing and human clinical studies. Indeed, Nasodine was tested in a sensitive human nasal epithelial cell model and shown to have no ciliotoxic or other cytotoxic potential up to 30 min exposure (21). Subsequently and prior to the current study, there have been four clinical studies of Nasodine:

1. Phase I safety study in healthy adult volunteers (22): concluded that there was no clinically significant iodine absorption and no impact on thyroid function from the use of Nasodine four times daily for five consecutive days; the nasal spray was well tolerated.
2. Phase II pilot study in COVID-19 subjects (23): concluded that the nasal spray was rapidly virucidal to SARS-CoV-2 *in vitro* using exposure times consistent with nasal residence, and that single *in vivo* nasal administration reduced infectious viral titers in COVID-19 subjects with culturable virus.
3. Phase II randomized controlled study in COVID-19 subjects (12): concluded that 20 doses of the nasal spray administered over two and a half days significantly reduced nasal shedding of viable SARS-CoV-2 virus with 100% clearance of viable virus from the nasal passages on the day after completion of treatment.
4. A Phase II pilot study (unpublished) was conducted in 39 subjects with URTI symptoms to test and refine a study design and tools for the current Phase III study. Based on the pilot study, the Phase III dose was set at three sprays per nostril (0.84 mL total dose), several refinements to the WebApp were made, and other trial parameters established.

The aim of this study was to assess the potential clinical benefits of a 0.5% w/v PVP-I nasal spray (Nasodine) as a treatment for the common cold.

## Materials and methods

### Ethics

This Phase III trial was conducted in accordance with the *International Council for Harmonisation (ICH) Guideline for Good Clinical Practice ICH E6 (R2)* and the *National Standard Operating Procedures for Clinical Trials* in Australia (ACTRN12619000764134). The study protocol and Participant Information and Consent Form (PICF) were approved by an independent human research and ethics committee.

### Study design

The study was a multi-center, randomized, double-blind, placebo-controlled study of the impact of Nasodine as a treatment for the common cold in adults. The study was managed by an independent clinical research organization (CRO).

Subjects: Two hundred and sixty (260) adults with common cold symptoms were recruited at two sites in two states in Australia during the 2019 common cold season (June-October 2019). For enrollment, subjects were required to be between 18 and 65 years old, have had cold symptoms for less than 60 h prior to enrollment, to report at least two of four key cold symptoms – sneezing, runny nose, nasal congestion, or sore throat—and have a Jackson score (24) of at least 3 points. They were also required to have a diagnosis of “common cold” confirmed by a medical officer at enrollment. Specific exclusion criteria were:

– Fever > 38°C or abnormal vital signs.
– Known iodine sensitivity.
– Known thyroid disease.
– Known immunodeficiency.
– Chronic respiratory diseases, including asthma, chronic cough, COPD, chronic allergic rhinitis or otherwise using chronic inhaled corticosteroids.
– Pregnant or nursing (lactating) or planning to become pregnant during the study.
– A doctor’s diagnosis in the previous 48 h of allergic rhinitis, bacterial sinusitis or lower respiratory tract infection.
– Taking any prescription medication that could affect assessment of the investigational product, as determined by a medical officer.
– Intending to use during the study any OTC cold medications that could influence study results including a povidone-iodine gargle; paracetamol was available as a rescue medication for any disabling symptoms.
– Unwilling to sign the informed consent form (PICF).

Inclusion/exclusion criteria data were collected at each study site by study personnel prior to enrollment and reported for each subject in the study eCRF. Eighty-six (86) subjects or 33% of total subjects enrolled were male and 174 subjects or 67% of total enrolled subjects were female. The mean age for females was 32.4 years and for males, 32.0 years. Based on race, 222 or 85% of all subjects were classified as White, while 12% were Asian.

Materials: The placebo for the study was a saline nasal spray (SNS), colored with an approved inert dye to match the Nasodine color and presented in an identical 25 mL bottle to Nasodine with non-removable identical spray pumps attached. Nasodine and SNS placebo were manufactured in a GMP facility that recorded the production process, release testing and batch numbering. Batches of Nasodine and placebo bottles were supplied to a contract clinical trial product manufacturer for labeling in accordance with the randomization schedule after which they were delivered directly to the clinical trial sites.

Blinding: The study was blinded for all investigators and subjects. Enrolled subjects were randomized 2:1 between Nasodine and placebo. The randomization schedule was prepared by the study statistician who nominated either Nasodine or placebo against sequential numbers, 10,001 to 10,258 for site 1 and 20,001 to 20,258 for site 2. Labels bearing these numbers were affixed to Nasodine or placebo bottles by the clinical trial product manufacturer. These labeled bottles were then provided to the trial sites and bottles issued to subjects as they were enrolled in bottle number order. Unblinding of the randomization schedule was not done until after database lock, before which only the statistician or one unblinded pharmacy monitor were aware of the allocation of the investigational product. This process ensured that site staff, clinicians, subjects, study management and sponsor were unaware of the allocation of investigational product to the subjects during the trial. To assess blinding effectiveness, all subjects were asked after their first dose whether they believed they had received the active product or placebo or were unsure. To avoid the effects of therapy on the answer, this question was asked only after the first supervised administration on the day of enrollment.

Powering: Based on subjects randomized 2:1 for Nasodine: placebo, a sample size of 255 was determined to have 80% power to detect a difference of 25% between the 5-day (Days 2–6) mean scores for Nasodine and placebo on the selected primary endpoint (NSS), using a *t*-test with a 5% 2-sided level of significance. The power calculations were derived from a previous 39-subject Phase II pilot study. Coincidentally, the current study was also powered for GSS as the primary endpoint. This was determined based on powering calculations used to design the earlier Phase II study, which was abridged to 39 subjects but originally planned and powered as a larger scale study involving 258 subjects with GSS as the primary endpoint.

Nasodine and placebo subjects were scheduled to each receive a total of 20 doses of nasal spray. Doses were self-administered four times daily over five days. Each dose was 6 actuations (three sprays in each nostril), equivalent to a total volume of 0.84 mL per dose.

### Outcome measures

**FIGURE 1:**
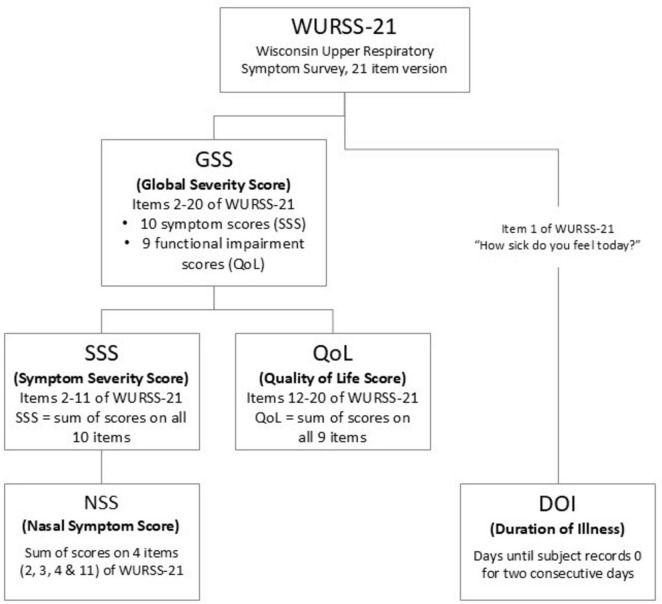
Structure and relationships of WURSS-21 outcome measures.

**FIGURE 2:**
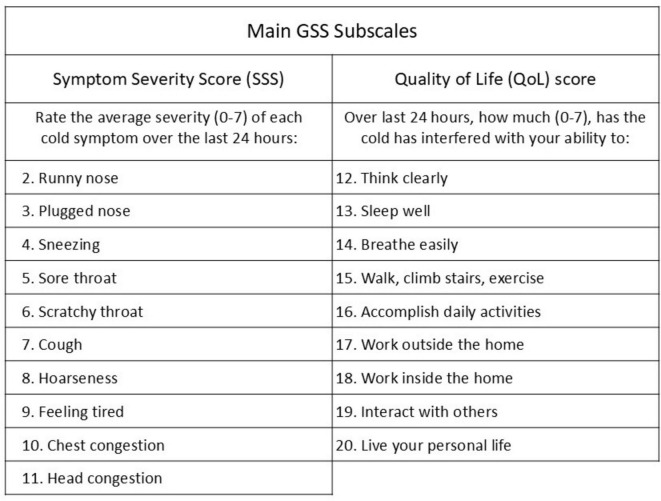
Individual items within SSS and QoL subscales of GSS.

All key outcome measures were derived from the Wisconsin Upper Respiratory Symptom Survey-21 (WURSS-21), which is a validated common cold patient reported outcome measure (PROM). The survey records 21 items. The validated outcome measure is the 19-item Global Severity Score (GSS), which is a measure of overall cold severity. The GSS is comprised of two subscales: (1) 10 items that measure common cold symptoms (Symptom Severity Score, SSS) and (2) nine items that measure functional impairment (QoL) as shown in Figures 1, 2. To evaluate the impact on nasal symptoms alone, the study included a Nasal Symptom Score (NSS), which was an unvalidated subscale intended to reflect nasal symptoms alone and comprised items 2, 3, 4 and 11 (runny nose, plugged nose, sneezing and head congestion) from the SSS scale (refer to Figure 2). Duration of illness (DOI) was a secondary endpoint. This was based on item 1 in the WURSS-21 survey, referred to as the global illness score (“How sick do you feel today?”).

The 4-item NSS scale was selected as the primary endpoint for the study, based on the Phase II pilot study, the results of which indicated that the principal effect of the topical nasal spray would be on nasal symptoms. Accordingly, the selected primary endpoint was the difference between Nasodine and SNS placebo on the mean NSS value (based on the Least Squared Mean, LSM) for Days 2–6 with Day 1 included as a covariate in the model.

Secondary endpoints included the difference between Nasodine and SNS on GSS for Days 2–6 based on LSM values, and SSS, similarly based on LSM values for Days 2–6, in both cases using Day 1 values as a covariate.

Duration of illness (DOI) was also included as a secondary endpoint and calculated as the mean number of days required for subjects to record zero (“Not sick”) for two successive days on item 1 of the WURSS-21 survey.

### On-study monitoring

WURSS-21 scores were reported once daily by each subject through a phone-based WebApp configured specifically for the study and registered by all subjects at enrollment on Day 1. Enrolled subjects recorded baseline WURSS-21 scores in the WebApp and then self-medicated with the assigned nasal spray (Nasodine or SNS) under supervision for their first dose. Subsequently, subjects left the clinic and were scheduled to continue unsupervised dosing four times daily for a total of 20 doses over the next five days, typically spanning six calendar days. The WebApp notified each subject to take and record each dose, and once daily, to complete the WURSS-21 questionnaire.

Subjects were questioned at each site visit and through the WebApp, from screening until the end of the study, about any adverse events (AEs) they experienced. They were questioned after each dose about any AEs and specifically prompted to report any (a) nasal stinging or burning, (b) throat stinging or burning, (c) headache or (d) dizziness, each of which had been identified as potential AEs in the pilot study. All AEs were documented in the eCRF, including the date of onset, a description of the AE, its severity and duration, actions taken and any administration of other treatments, outcomes and an investigator’s opinion on the relationship between the treatment and the event.

### Viral efficacy sub-study

Seventy-three randomized subjects participated in a viral identity and shedding sub-study: samples were collected at the clinical study sites by instillation of a small volume of saline (280 μL) into each nostril, then within 30 s, nose-blowing onto a suitable area of plastic wrap, from which a swab sample (in duplicate) was immediately collected by site staff. Duplicate swabs were collected at baseline (Day 1) prior to treatment and then once daily on Days 2, 3, 4, 7, and 14. Swabs were tested for virus identity and presence or absence of virus against a PCR-based respiratory pathogen panel. The mean time from enrollment to cessation of viral shedding (TCS) in the sub-study was included as an exploratory endpoint.

### Statistical analysis

For efficacy assessments against the primary and secondary endpoints (other than DOI), a general linear model (GLM) was fitted to the data with mean total score over Days 2–6 as the analysis variable and treatment and site as factors. Day 1 (baseline) scores were included as a covariate. From the model the least squares means (LSMs) were obtained (with 95% confidence limits) for each treatment group. In addition, the difference in LSMs (placebo—Nasodine) was obtained together with 95% confidence limits and the *p*-value calculated. The null hypothesis tested was that there is no difference in LSMs between treatments against the two-sided alternative hypothesis that there is a difference between means.

## Results

### Enrollments

**FIGURE 3:**
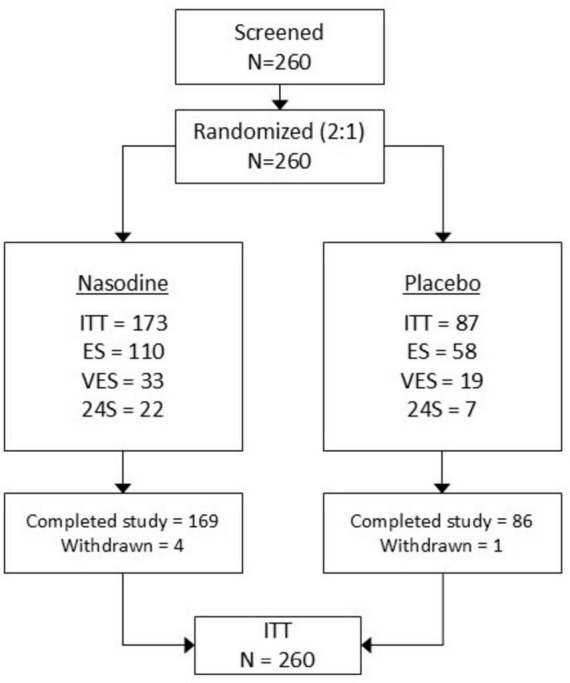
Subject disposition.

The disposition of all subjects enrolled in the study is summarized in Figure 3. A total of 260 subjects were screened and randomized and all were included in the ITT analysis. After unblinding, it was determined that 173 received Nasodine and 87 received SNS. Five subjects were withdrawn from the study (no withdrawals were due to treatment related adverse effects) at some point after starting their treatment. The demographics of enrolled subjects are summarized in Table 1. Population subsets for analysis that were defined *post hoc* were as follows:

• ES: The Efficacy Subset (ES) comprised 168 subjects in the ITT whose baseline symptoms were rated stronger than “mild.”
• VES: The Viral Efficacy Subset (VES) comprised the 52 subjects in the ITT subset who had laboratory confirmed viral colds (primarily human rhinoviruses and seasonal coronaviruses).
• 24S: The subset of 29 subjects who commenced treatment within 24 h after symptom onset.

**TABLE 1:** Demographic and baseline characteristics (ITT population).

	SNS placebo (*N* = 87)	Nasodine (*N* = 173)
Age (years); mean (SD)	32.4 (13.56)	32.0 (13.23)
**Gender; n (%)**		
Male	26 (29.9%)	60 (34.7%)
Female	61 (70.1%)	113 (65.3%)
**Race**		
White	73 (83.9%)	149 (86.1%)
Black/African American	2 (2.3%)	1 (0.6%)
Asian	9 (10.3%)	21 (12.1%)
Native Hawaiian/Other Pacific Islander	0 (0.0%)	2 (1.2%)
Aboriginal/Torres Strait Islander	1 (1.1%)	0 (0.0%)
Other	2 (2.3%)	0 (0.0%)
Body mass index (kg/m^2^); mean (SD)	26.11 (5.510)	26.59 (6.797)
Jackson score; mean (SD)	10.3 (3.27)	10.0 (3.32)

### Efficacy

**FIGURE 4:**
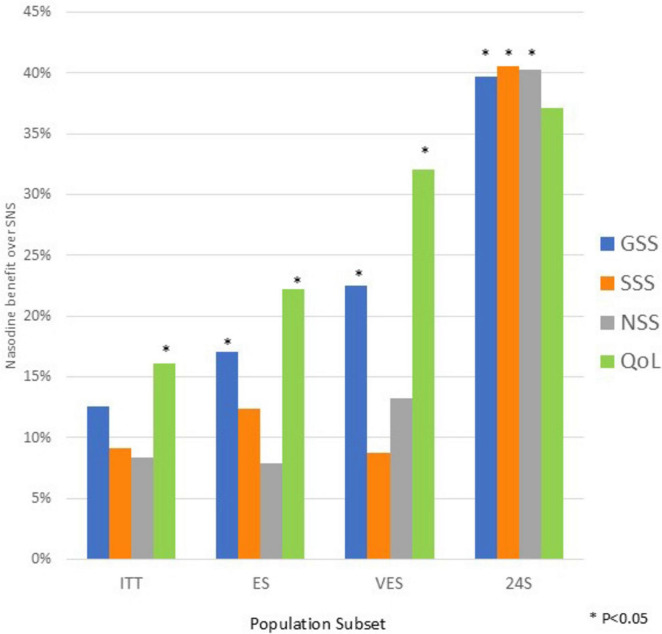
Summary of Nasodine benefit over SNS by GSS scales and treatment subsets. Based on data in Tables 2–5.

The efficacy results for the ITT and defined subsets are shown in Tables 2–5 and summarized in Figure 4. All the WURSS-21 outcomes trended in favor of Nasodine treatment compared with SNS placebo. In the ITT, the Nasodine GSS benefit over placebo (12.6%) approached statistical significance (*P* = 0.054). On the GSS subscales, the Nasodine benefit on SSS was 9.1% (*P* = 0.169) and on NSS was 8.4% (*P* = 0.217). The benefit on the QoL scale was 16.1% and was statistically significant (*P* = 0.041).

**TABLE 2:** Difference in mean total GSS between treatments from the GLM.

Population	Subjects (Placebo/Nasodine)	Placebo LSM (95% CL)	Nasodine LSM (95% CL)	Difference LSM (95% CL)	Nasodine benefit *p*-value
ITT	*N* = 260 (87/173)	36.13 (32.36, 39.90)	31.58 (28.91, 34.25)	4.55 (−0.08, 9.17)	12.6% *p* = 0.0539
**ES**	***N* = 168** **(58/110)**	**40.44** **(35.62, 45.27)**	**33.51** **(30.01, 37.01)**	**6.93** **(0.96, 12.90)**	**17.1%** ***p* = 0.0231**
**VES**	***N* = 52** **(19/33)**	**45.56** **(37.58, 53.54)**	**35.33** **(29.33, 41.33)**	**10.23** **(0.08, 20.37)**	**22.5%** ***p* = 0.0482**
**24S**	***N* = 29** **(7/22)**	**42.91** **(30.42, 55.40)**	**25.87** **(19.08, 32.66)**	**17.04** **(2.47, 31.62)**	**39.7%** ***p* = 0.0241**

Bold figures indicate a statistically significant result at *p* < 0.05. Data presented are: Least squares mean (LSM) of total score Days 2–6 (95% Confidence limits). “Nasodine Benefit” is calculated as the Difference divided by the placebo. P-value is based on the difference between placebo and Nasodine LSMs.

**TABLE 3:** Difference in mean total SSS between treatments from the GLM.

Population	Subjects (Placebo/Nasodine)	Placebo LSM (95% CL)	Nasodine LSM (95% CL)	Difference LSM (95% CL)	Nasodine benefit *p*-value
ITT	*N* = 260 (87/173)	17.73 (15.86, 19.60)	16.12 (14.79, 17.44)	1.61 (−0.69, 3.90)	9.1% *p* = 0.1686
ES	*N* = 168 (58/110)	19.87 (17.42, 22.32)	17.41 (15.63, 19.18)	2.46 (−0.57, 5.49)	12.4% *p* = 0.1112
VES	*N* = 52 (19/33)	20.29 (16.59, 23.99)	18.51 (15.73, 21.29)	1.78 (−2.93, 6.49)	8.8% *p* = 0.4518
**24S**	***N* = 29** **(7/22)**	**25.24** **(18.33, 32.16)**	**15.00** **(11.26, 18.75)**	**10.24** **(2.16, 18.32)**	**40.6%** ***p* = 0.015**

Bold figures indicate a statistically significant result at *p* < 0.05. Data presented are: Least squares mean (LSM) of total score Days 2–6 (95% Confidence limits). “Nasodine Benefit” is calculated as the Difference divided by the placebo. *P*-value is based on the difference between placebo and Nasodine LSMs.

**TABLE 4:** Difference in mean total NSS between treatments from the GLM.

Population	Subjects (Placebo/Nasodine)	Placebo LSM (95% CL)	Nasodine LSM (95% CL)	Difference LSM (95% CL)	Nasodine benefit *p*-value
ITT	*N* = 260 (87/173)	7.52 (6.70, 8.34)	6.89 (6.31, 7.47)	0.63 (−0.37, 1.63)	8.4% *p* = 0.2168
**ES**	***N* = 168** **(58/110)**	7.35 (6.55, 8.16)	6.78 (6.19, 7.36)	0.58 (−0.42, 1.57)	7.9% *p* = 0.2555
**VES**	***N* = 52** **(19/33)**	8.54 (7.47, 9.62)	7.41 (6.63, 8.19)	1.13 (−0.19, 2.46)	13.2% *p* = 0.0936
**24S**	***N* = 29** **(7/22)**	**11.07** **(8.23, 13.90)**	**6.61** **(5.06, 8.15)**	**4.46** **(1.16, 7.76)**	**40.3%** ***p* = 0.010**

Bold figures indicate a statistically significant result at *p* < 0.05. Data presented are: Least squares mean (LSM) of total score Days 2–6 (95% Confidence limits). “Nasodine Benefit” is calculated as the Difference divided by the placebo. P-value is based on the difference between placebo and Nasodine LSMs.

**TABLE 5:** Difference in mean total QoL between treatments from the GLM.

Population	Subjects (Placebo/Nasodine)	Placebo LSM (95% CL)	Nasodine LSM (95% CL)	Difference LSM (95% CL)	Nasodine benefit *p*-value
**ITT**	***N* = 260** **(87/173)**	**18.44** **(16.12, 20.76)**	**15.47** **(13.83, 17.12)**	**2.96** **(0.12, 5.81)**	**16.1%** ***p* = 0.0414**
**ES**	***N* = 168** **(58/110)**	**20.69** **(17.79, 23.59)**	**16.09** **(13.99, 18.20)**	**4.59** **(1.01, 8.18)**	**22.2%** ***p* = 0.0124**
**VES**	***N* = 52** **(19/33)**	**24.99** **(19.58, 30.41)**	**16.98** **(12.89, 21.07)**	**8.02** **(1.18, 14.85)**	**32.1%** ***p* = 0.0225**
24S	*N* = 29 (7/22)	17.41 (10.87, 23.95)	10.95 (7.37, 14.52)	6.46 (−1.16, 14.08)	37.1% *p* = 0.093

Bold figures indicate a statistically significant result at *p* < 0.05. Data presented are: Least squares mean (LSM) of total score Days 2–6 (95% Confidence limits). “Nasodine Benefit” is calculated as the Difference divided by the placebo. *P*-value is based on the difference between placebo and Nasodine LSMs.

In population subsets, the magnitude of Nasodine benefits over placebo on GSS and most subscales increased in subjects with stronger cold symptoms at enrollment (ES), those with laboratory confirmed viral infection (VES) and those who started treatment within 24 h of symptom onset (24S).

**FIGURE 5:**
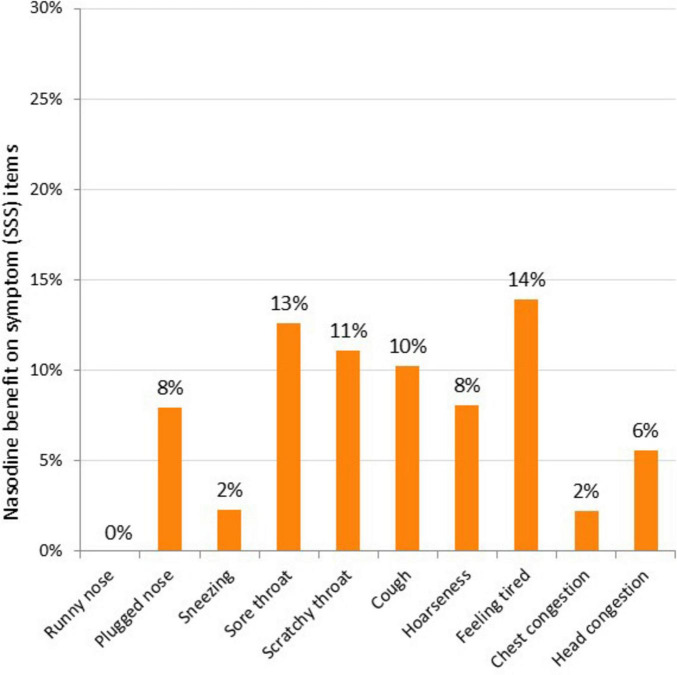
Nasodine benefit over SNS on individual SSS items in ITT. Benefit measured as the difference between Nasodine and SNS subjects in mean total scores for Days 2–6, divided by the mean total score for SNS subjects for Days 2–6.

**FIGURE 6:**
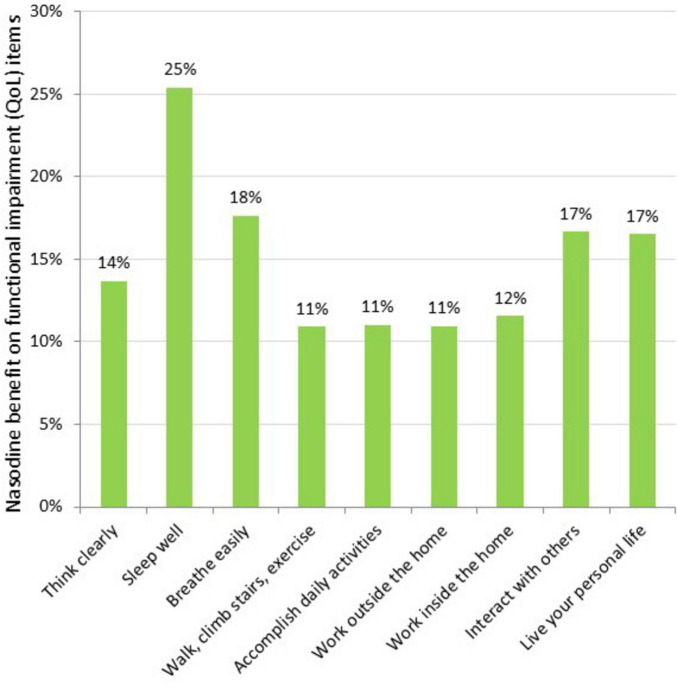
Nasodine benefit over SNS on individual QoL items in ITT. Benefit measured as the difference between Nasodine and SNS subjects in mean total scores for Days 2–6, divided by the mean total score for SNS subjects for Days 2–6.

To further characterize the clinical effects of Nasodine, a *post hoc* analysis of the results on each of the 19 GSS items was conducted. The average benefit (severity score reduction) of Nasodine versus placebo was calculated for each of the individual SSS and QoL items (Figures 5, 6). No statistical analysis was conducted on the individual item differences. This analysis revealed a positive benefit on 18 of the 19 GSS items, with the greatest impact seen on QoL items, such as impact on ability to “sleep well,” “breathe easily,” “interact with others,” and “live your personal life.”

In terms of duration of illness, the median DOI was 10.0 days for both treatments (95% confidence limits 9.0–11.0 for both treatments), suggesting no difference between treatments on this measure (Table 6).

**TABLE 6:** Results for analysis of duration of illness (DOI) in ITT.

	Overall (*N* = 260)	Placebo (*N* = 87)	Nasodine (*N* = 173)
**Illness resolved**			
No, n (%)	72 (27.7%)	23 (26.4%)	49 (28.3%)
Yes, n (%)	188 (72.3%)	64 (73.6%)	124 (71.7%)
95% Confidence limits (CI)	66.4%, 77.7%	63.0%, 82.4%	64.3%, 78.3%
Median duration (days) (95% CI)	10.0 (9.0, 10.0)	10.0 (9.0, 11.0)	10.0 (9.0, 11.0)
Hazard ratio, 95% CI (from Cox PH model)	1.009 (0.741, 1.359) *p* = 0.956		

In relation to the exploratory endpoint, the median time to cessation of viral shedding (TCS) was three days sooner in the Nasodine arm compared with the placebo arm. However, this difference was not statistically significant (*P* = 0.42).

For the 52 subjects (71% of those sampled) where a virus was detected, human rhinovirus (HRV) was the most common virus (55% of detections), followed by seasonal coronaviruses (27%), Human metapneumovirus (8%), respiratory syncytial virus (8%) and parainfluenza virus (2%). No virus was detected by PCR in 29% of the 73 sampled subjects.

There was good compliance overall. Major protocol deviations were reported in 14 subjects: 12 from the Nasodine arm and two from the placebo arm, due to failure to provide WURSS-21 scores on study days 2 through 6, failure to take an adequate dose (<50% of the intended dose target) or other reasons. Five subjects were withdrawn from the study: one subject from the placebo arm due to an adverse event, two subjects from the Nasodine arm due to adverse events. None of the study withdrawals were considered related to the study drug.

### Safety and tolerability

There were no serious adverse events (SAE) and the majority of treatment emergent adverse events (TEAE) reported were classified as mild. The TEAEs are shown in Table 7. Only rhinalgia was found to be significantly more prevalent in Nasodine recipients compared to placebo (RR 5.364; 95% CL 2.529, 11.82, *p* < 0.0001). Rhinalgia (manifested as nasal irritation) after application was reported at least once by approximately 37% of subjects across their scheduled 20 doses, compared to 7% for placebo. It was rated mild in 83% of cases, and in all cases was transient. In no case was rhinalgia a cause of discontinuation of treatment or withdrawal from the study. This finding was consistent with previous clinical studies. Overall, Nasodine treatment was well tolerated with no evidence of any significant adverse effects or compliance concerns.

**TABLE 7:** Number and percentage of subjects experiencing TEAE by MedDRA preferred term (at least five subjects with events with the PT shown).

MedDRA preferred term Data displayed: subjects (%)	Placebo (*N* = 87)	Nasodine (*N* = 173)	All patients (*N* = 260)
Rhinalgia	6 (6.9%)	64 (37.0%)	70 (26.9%)
Headache	18 (20.7%)	36 (20.8%)	54 (20.8%)
Oropharyngeal pain	5 (5.7%)	17 (9.8%)	22 (8.5%)
Epistaxis	5 (5.7%)	13 (7.5%)	18 (6.9%)
Dizziness	4 (4.6%)	9 (5.2%)	13 (5.0%)
Throat irritation	2 (2.3%)	10 (5.8%)	12 (4.6%)
Nasal discomfort	0 (0.0%)	8 (4.6%)	8 (3.1%)
Rhinorrhea	1 (1.1%)	6 (3.5%)	7 (2.7%)
Nausea	4 (4.6%)	3 (1.7%)	7 (2.7%)
Nasal congestion	0 (0.0%)	6 (3.5%)	6 (2.3%)
Sneezing	1 (1.1%)	5 (2.9%)	6 (2.3%)
Lacrimation increased	2 (2.3%)	4 (2.3%)	6 (2.3%)
Sinus congestion	2 (2.3%)	3 (1.7%)	5 (1.9%)

Subjects are counted only once in each row, i.e., multiple events mapping to the same subject are counted only once.

The risk of iodine-related staining of skin or clothing was not found to be a concern. Staining was not reported by participants or investigators.

### Blinding effectiveness

After their first post-enrollment dose, subjects were asked whether they thought they had received the active, placebo or were unsure. Post-trial analysis of the results of this question indicated that acceptable blinding had occurred: most subjects stated they were unsure (53% of active and 62% of placebo recipients); 40% of active recipients and 29% placebo recipients believed they had received the “active” product; and 6% of active recipients and 9% of placebo recipients believed they had received the placebo. There were no significant differences between active and placebo recipients in any group.

## Discussion

Nasodine was well tolerated when applied at a dose of three sprays per nostril, four times daily over five days. The most common adverse events were related to transient nasopharyngeal discomfort. These findings are consistent with the other clinical studies of Nasodine where higher doses were used (four sprays per nostril) (12, 22, 23).

This Phase III clinical trial provides a rich pool of efficacy data offering a breadth of insights into the performance of PVP-I nasal spray as a treatment for the common cold. The dataset included severity scores collected over five days for 260 subjects, each reporting once-daily on 19 common cold items that aggregate to the GSS, which is a validated measure of overall cold severity provided by the WURSS-21 questionnaire.

The previously conducted 39-subject pilot Phase II study results indicated that the impact of Nasodine appeared to be greatest on nasal symptoms. Consequently, the NSS subscale was selected as the primary end point for the Phase III study. However, after unblinding and analysis in the current study, it became clear that the hypothesis that Nasodine acted primarily on nasal symptoms was ill-informed and that the impact of the treatment was broader and more clinically compelling than just an effect on local symptoms, as indicated by Figures 4–6. This warranted a broader investigation into the clinical findings rather than focusing on the pre-stated primary endpoint as the measure of clinical efficacy.

The use of SNS as the placebo likely suppressed the reported benefit of Nasodine over placebo on the 4-item nasal symptom score, and consequently, the modest reported benefit on the 10-item symptom severity score (SSS). The use of SNS and the overall results of the study also need to be framed against the fundamental challenges of conducting a common cold clinical study. These include:

1. *Enrollment:* Enrolling subjects early enough in the cold symptom cycle to discern an effect in a relatively short-lived and self-resolving condition is a significant challenge in a natural setting study. This is particularly important for an intervention that seeks to interrupt the complex, immune-response-mediated, symptom pathway by suppressing viral load, which by around 48 h after symptom onset, is already declining due to the immune response. Subjects here were recruited up to 60 h after symptom onset, with a mean of 40 h post onset. Commencing treatment within 24 h after symptom onset is crucial to achieving maximum benefit.
2. *No objective endpoint:* Patient reported outcome measures (PROMs) such as the WURSS-21 are subjective and affected by multiple psychological and situational factors, making them prone to bias. Ideally, they should be used in conjunction with objective outcomes, but currently there are no objective outcome measures for the common cold (25).
3. *Placebo:* Nasal administration of a topical antimicrobial agent makes defining an appropriate placebo challenging, while maintaining blinding goals. Apart from a placebo effect, any placebo nasal solution may introduce mechanical, dilutionary or other physicochemical effects that could have some impact on symptoms. This means that most, if not all, are not true placebos. SNS was selected as the placebo in this trial. Numerous studies indicate that a SNS has a positive effect on local cold symptoms (26–30). Indeed, these products are marketed for relief of local symptoms associated with the common cold in many countries worldwide. Although saline may have had a positive impact, no comparable WURSS-21 data were available in the public domain which could be used to calibrate the extent of the “placebo” activity in the present dose form. Indeed, Ramalingam et al. (30) who recently reported that saline nasal irrigation and gargling resulted in significant reduction in the duration of nasal symptoms, cough and hoarseness in common cold subjects, concluded that until a safe and comfortable placebo is identified, placebo-controlled trials for nasal administered agents in the common cold cannot be done.
4. *Symptom severity at baseline*: The study protocol allowed the inclusion of subjects with very mild symptoms at baseline (Jackson score of 3). This makes it statistically more difficult to detect an efficacy signal based on symptom reduction. The ES subset, which comprised 65% of ITT (168 subjects) who had stronger symptoms at enrollment (at least one symptom reported as at least “moderate” rather than “mild”), are relevant in this regard. From a practical perspective, they are also relevant given that those with stronger symptoms are more likely to be motivated to use treatment.

Against the background of these challenges, the 12.6% difference (*P* = 0.054) in favor of Nasodine over SNS on GSS in the ITT is considered clinically meaningful. Underpinning this view is the fact that GSS benefit became greater, and in all cases statistically significant, as relevant population subsets were considered. In the ES population of subjects who had higher symptom scores at enrollment, the difference in GSS increased to 17.1% (*P* = 0.023). In the VES subset, comprising subjects who had laboratory confirmed viral infections, which are particularly relevant from a clinical proof of concept perspective, the GSS benefit over SNS increased to 23.0% (*P* = 0.048). Finally, in those subjects who initiated treatment within 24 h of symptom onset, the difference in GSS increased to 39.7% (*P* = 0.024), despite the small number of subjects (*N* = 29).

The impact on the QoL subscale was far more pronounced than on the SSS subscale. The QoL subscale measures the degree of functional impairment caused by colds and given the potential confounding effects of placebo on local symptoms, it may be a more valid and clinically relevant measure of the impact of the intervention on cold severity in this case. For QoL, the differences were all favorable for Nasodine and generally statistically significant. In the ITT, the QoL benefit was 16.1% in favor of Nasodine (*P* = 0.041); this increased to 22.2% in the ES (*P* = 0.012) and 32.1% in the VES (*P* = 0.023). In subjects with symptoms for less than 24 h, the benefit was 37.1% (*P* = 0.093). These results support a consistently positive effect on the degree of impairment caused by a cold.

Despite the favorable results on GSS and QoL, Nasodine had no discernible benefit over placebo in reducing cold duration (DOI) in the ITT. This could reflect how this outcome was measured, which was the time in days to reach a point where subjects reported they did not feel sick at all for two consecutive days, with the result being 10.0 days for both treatment groups. This may not be an ideal measure for a topical antimicrobial intervention that acts by suppressing the extracellular viral load, which may down-regulate the immune response that is driving symptoms but leave trailing symptoms through the normal course of a cold. In a *post hoc* analysis, a “time to alleviation of illness” (TAI) was defined as the time in days to reach a point where no functional impairment (QoL) score was greater than “mild”. The TAI for Nasodine subjects was 3.4 days compared with 4.0 days for placebo subjects, a difference that was statistically significant (*P* = 0.007).

There are several limitations in the study. The elevation of GSS from secondary endpoint to primary endpoint after completion of the study departs from convention. However, this is justified for several reasons: (a) the study was powered for GSS (as well as NSS); (b) the 19-item GSS is the validated outcome of the WURSS-21, while the 4-item NSS is not a validated scale; (c) the NSS is part of the GSS, but the 19-item GSS is a statistically richer measure than its 4-item subscale, and (d) in hindsight, NSS did not meet the definition of a primary endpoint in that it did not “fully characterize clinically the effect of a treatment” (31), whereas GSS did. Indeed, NSS should not have been selected initially as the primary endpoint. However, elevation of GSS to primary endpoint, while justified, potentially introduces statistical “multiplicity” and increases the risk of Type 1 error (erroneously rejecting the null hypothesis). The standard approach for addressing multiplicity is a Bonferroni correction, but this was inappropriate given the high correlation between GSS and NSS, given that latter is a subset of the former. An alternative adjustment model that considered the correlation resulted in an adjusted *P*-value for the Nasodine benefit in the ITT of 0.085 compared with the original value of 0.054.

The impact of the SNS placebo also needs further consideration. An analysis of the potential impact using the study dataset revealed that if the SNS had conservatively reduced the value of the placebo GSS score by only 1.5% (0.55 units), such that the “true” placebo mean GSS score was 36.68 instead of 36.13, the *P*-value associated with the Nasodine benefit would reduce from 0.085 to 0.049, i.e., a 1.5% saline effect would increase the Nasodine GSS benefit in the ITT from 12.6 to 13.9% and make the outcome statistically significant, even after adjustment for multiplicity. As noted above, numerous studies have pointed to the clinical efficacy of intranasal saline in the common cold, and there is a multitude of saline nasal products marketed for relief of local cold symptoms. In the case of the current study, the conclusion that the SNS placebo was likely an active comparator would be justified and that its effect was likely much greater than the 1.5% required to arrive at a statistically significant GSS benefit for Nasodine in the ITT, even after a multiplicity adjustment.

Regardless of the statistics, the question remains whether a saline-adjusted benefit of 13.9% is “clinically meaningful.” Because there is no established regulatory definition for what constitutes a clinically meaningful treatment for the common cold, this is subjective. The following issues should be considered to address this question:

(a) There is no objective measure for efficacy in the common cold, and the only available and validated measures are patient-reported outcome measures (PROMs) such as the WURSS-21; such tools are blunt instruments and any statistically significant benefit, however, modest, might be considered clinically meaningful.
(b) SNS likely was an active comparator and is widely used for the alleviation of cold symptoms; therefore, any benefit over SNS, even a small one, could be considered meaningful.
(c) The GSS benefits over SNS (without adjustment) in the ES, VES and 24S subsets were substantial and significant, approaching 40% in the 24S subset; in particular, the QoL benefits would be evaluated as very meaningful by a cold sufferer.
(d) The study recruited subjects on average 40 h after symptom onset; in the real world, cold sufferers would seek treatment much sooner and based on the response of subjects who were recruited within 24 h of symptom onset, they would likely achieve highly meaningful clinical outcomes.

Our view is that despite all the challenges—the PROM, recruitment on average 40 h after symptom onset, and the suppressive effect of an active placebo—Nasodine demonstrated a consistently positive clinical benefit on all cold severity outcomes and subscales. In many instances, the results were also statistically significant. These findings, especially relating to functional impairment, are considered clinically meaningful.

The trial had other limitations related to its inclusion-exclusion criteria. It did not include sites outside Australia, children, people with common conditions such as thyroid disease and asthma, or anyone taking a cold medication. These exclusions might raise questions about the generalizability of the trial’s findings.

Generalizability concerns often arise with systemically acting agents where the efficacy and safety of a specific drug can depend on pharmacokinetic and pharmacodynamic factors, including absorption, bioavailability, distribution, metabolism and excretion, in addition to receptor binding, post-receptor effects and interactions with other drugs. These factors can vary between populations, potentially of the types excluded in the current trial. However, none of these factors is conceivably relevant for a topical broad-spectrum agent like PVP-I.

The groups excluded from the trial were excluded for conservative safety reasons or because they might confound subject reporting. However, it is unlikely that any of those excluded would experience a different efficacy outcome from the topical intranasal use of Nasodine in treating an URTI. Conceivably, the efficacy benefit from Nasodine could vary between people based on different local immune factors that might lead to different levels of experienced cold severity; for example, the elderly have fewer and less severe colds due to diminished immune responsiveness. However, those with fewer or milder colds may not feel the need to use Nasodine, or any cold medications for that matter, so the question may be moot in practice.

In terms of safety, there are some generalizability limitations. While the level of iodide absorption may be clinically insignificant in euthyroid adults, use in children is yet to be evaluated and long-term use in those with thyroid conditions may warrant limitation. Given the low level of iodine absorption, acute use in pregnancy and breastfeeding should not be a practical limitation, especially given the higher nutritional requirement for iodine. Clinicians should also consider the perspective that PVP-I gargles are widely used in many countries to treat sore throats and the level of available iodine in a single 15 mL dose of a 1% PVP-I gargle is approximately 30 times that of a single 0.84 mL dose of Nasodine.

An historical safety concern has been iodine allergy and subjects were excluded from the study if they reported they were allergic to iodine. However, a recent review of 81 papers in the field concluded that iodine has no inherent allergic potential (32) and free iodine is well-tolerated by human tissue, even at high concentrations (6). Reported iodine “allergy” appears to have arisen from observed reactions to shellfish and radiocontrast dyes; however, shellfish allergy has been attributed to tropomyosin (a muscle protein) and contrast dye allergy to a hyperosmolar reaction (33). No allergic or sensitivity reactions to Nasodine were observed in the current or studies of Nasodine, but reactions to PVP-I have been reported in the literature. These are now believed to be due to non-iodinated copolymers in PVP (34), although true anaphylactic reactions to PVP-I are considered exceptionally rare (35, 36).

In summary, topical intranasal PVP-I offers a low-risk approach to the management of URTI in adults, with few practical limitations from a clinical perspective. Importantly, in the current study, it was demonstrated that Nasodine 0.5% PVP-I nasal spray delivered clinically meaningful and generalizable efficacy benefits compared with saline nasal spray, which is widely used as a first line treatment for common cold symptoms.

The clinical implications are considerable: The common cold is the most common illness of humanity, with a massive aggregate disease burden and annual productivity cost. In the absence of any vaccine or antiviral treatment, sufferers resort to symptomatic cold medications that are costly, have no effect on the viral cause of the illness and carry potential for adverse effects. Further, too often consumers are prescribed antibiotics that have no benefit and exacerbate the global problem of antimicrobial resistance.

Intranasal PVP-I represents a novel low-risk approach to the clinical management of URTI. It is the first intervention in URTI that targets the microbial cause of the illness. As a topical treatment, it also has few usage limitations and virtually no safety downside. It also offers clinicians an effective tool to deflect requests for antibiotics from patients with URTI.

The current study demonstrated for the first time that targeting the viral load in the nose with a 0.5% PVP-I nasal spray (Nasodine) has a beneficial effect on cold severity and especially functional impairment. It may be intuitively sensible that a local, broad-spectrum, microbicidal agent would be a useful intervention for URTI, but this is the first study to demonstrate the fact.

Based on the same mode of action and similar intuition, the clinical utility of a PVP-I nasal spray could extend beyond treatment of URTI. Recently, it was proposed that intranasal PVP-I could play a role in the management of pandemics based on PVP-I’s known activity against highly pathogenic viruses, specifically including Ebolaviruses, SARS and MERS coronaviruses and avian influenza viruses (37). A recent study showed that Nasodine significantly reduced nasal shedding of SARS-CoV-2 virus from COVID-19 patients (12, 38).

Similarly, it seems reasonable to conclude that the use of a PVP-I nasal spray would result in reduced viral shedding during an URTI. This could reduce the risk of transmission of the illness to others. By suppressing the intranasal viral load during an URTI, PVP-I nasal spray could also reduce the risk of patients’ aspirating virus from their nasal passages into their lungs. This could help reduce the risk of secondary illnesses for all patients, but importantly, it could help lower the risk of respiratory exacerbations for asthmatics and life-threatening LRTI in the elderly, immune-compromised, cystic fibrosis and COPD patients, and others with compromised lung function.

In the hospital setting, the clinical utility of a PVP-I nasal spray could readily extend to hospital infection control, where it could play a role in nasal decolonization of potentially pathogenic bacteria (PPB) (39). This could have value in reducing the risk of surgical site infections and augment (or replace) the use of intranasal mupirocin ointment, which may be prone to bacterial resistance. Aspiration of PPB from the upper airway is also a cause of bacterial pneumonia and intranasal PVP-I has been proposed as a countermeasure.

Given the broad potential clinical utility of intranasal PVP-I, along with its excellent safety profile, low cost, ease of use and easy deployability, its availability should not be unduly constrained by regulatory hurdles that are designed primarily for systemic drugs with narrow therapeutic indications.

The current study attempted to demonstrate its clinical utility in one indication—treatment of the common cold in adults—applying contemporary standards and methods of clinical design and statistical analysis, that were intended for systemically-acting therapeutics. From a regulatory perspective, the common cold is an acutely difficult indication for approval: there is no objective measure for efficacy; PROMs present the potential for error and bias and there is no universal agreement as to an acceptable PROM; there is no standard of care; there is no accepted level of clinical benefit that is “clinically meaningful”; and for an intranasal product, any intranasal placebo can confound efficacy measures. Therefore, it is not surprising that no new effective treatment for the common cold has been approved by major regulatory bodies, like the US Food and Drug Administration (FDA), in over 50 years.

Despite all these challenges, the study showed that Nasodine outperformed SNS especially on functional impairment, providing important proof that targeting the viral cause of URTI with a topical microbicidal agent can have a therapeutic benefit; and that it is a legitimate strategy for novel and safe intervention in the world’s most common illness for which there is no effective treatment. However, under the regulatory requirements in most advanced nations, this single trial may not be adequate for approval of topical PVP-I as a treatment for the common cold. Further, each of the other potential uses outlined above would be considered new indications and potentially require confirmatory clinical trials before the specific indication could be approved. Given the non-systemic mode of action of PVP-I and lack of potential for microbial resistance, its safety profile and broad potential clinical utility, a regulatory approach is called for that is less constrained by methods and pathways designed primarily for systemic drugs.

In conclusion, this was a multi-center, randomized, controlled, double-blind, Phase III clinical trial evaluating a 0.5% PVP-I nasal spray (Nasodine) for the common cold. The trial had several limitations which are addressed. These include the confounding effects of SNS as placebo, endpoint selection and associated multiplicity, as well as an array of challenges associated with any trial that attempts to assess a therapeutic intervention in a short-lived, acute illness, such as the common cold. It can be easy for these complications to cloud the important findings of this pivotal study and the broad potential for intranasal PVP-I.

In the current study, Nasodine 0.5% PVP-I nasal spray demonstrated a consistently positive benefit in reducing overall cold severity outcomes compared with saline nasal spray. The benefit of Nasodine was most evident in its impact on functional impairment (quality of life) scores, where the results were statistically significant and clinical meaningful. In subjects who started treatment within the first 24 h after symptom onset, the benefit of Nasodine over saline nasal spray was approximately 40%. It was safe and well tolerated. Nasodine is an effective and clinically meaningful treatment for the common cold.

## Data Availability

The original contributions presented in the study are included in the article, further inquiries can be directed to the corresponding author.
